# The Pre-Vaccination Donated Blood Is Free from Severe Acute Respiratory Syndrome Coronavirus-2 (SARS-CoV-2) but Is Rich with Anti-SARS-CoV-2 Antibodies: A Cross-Section Saudi Study

**DOI:** 10.3390/ijerph19127119

**Published:** 2022-06-10

**Authors:** Abdulrahman H. Almaeen, Abdulrahman A. Alduraywish, Amany A. Ghazy, Tarek H. El-Metwally, Mohammad Alayyaf, Fahad Hammad Alrayes, Ahmed Khalid M. Alinad, Saqer Bulayhid H. Albulayhid, Abdulrhman Rabea Aldakhil, Ahmed E. Taha

**Affiliations:** 1 Department of Pathology, College of Medicine, Jouf University, Sakaka 72388, Saudi Arabia; ahalmaeen@ju.edu.sa; 2 Internal Medicine Department, College of Medicine, Jouf University, Sakaka 72388, Saudi Arabia; dr-aaad@ju.edu.sa; 3 Microbiology and Immunology Division, Department of Pathology, College of Medicine, Jouf University, Sakaka 72388, Saudi Arabia; aaelshenawy@ju.edu.sa (A.A.G.); aeattia@ju.edu.sa (A.E.T.); 4 Department of Microbiology & Medical Immunology, Faculty of Medicine, Kafrelsheikh University, Kafrelsheikh 33516, Egypt; 5 Department of Pathology, Biochemistry Division, College of Medicine, Jouf University, Sakaka 72388, Saudi Arabia; thelmetwally@ju.edu.sa; 6 Department of Medical Biochemistry, Faculty of Medicine, Assiut University, Assiut 71517, Egypt; 7 Prince Mutaib Bin Abdulaziz Hospital, Sakaka 72388, Saudi Arabia; mohd_alayyaf@hotmail.com; 8 College of Medicine, Jouf University, Sakaka 72388, Saudi Arabia; 361100359@ju.edu.sa (F.H.A.); ahmedalenad@gmail.com (A.K.M.A.); 361100385@ju.edu.sa (S.B.H.A.); abdullr7man.77@gmail.com (A.R.A.); 9 Medical Microbiology and Immunology Department, Faculty of Medicine, Mansoura University, Mansoura 35516, Egypt or

**Keywords:** blood transfusion, COVID-19, SARS-CoV-2, anti-SARS-CoV-2 IgM, anti-SARS-CoV-2 IgG

## Abstract

(1) Backgrounds and Objectives: Since its discovery, information about the severe acute respiratory syndrome-coronavirus-2 (SARS-CoV-2) has spread rapidly. However, many issues remain unresolved. Coronaviruses are primarily transmitted through respiratory secretions. The possibility of transmission via donated blood transfusion deserves studying. This is the first study in Saudi Arabia to look at pre-vaccination donated blood anti-SARS-CoV-2 antibody content as a marker for virus transmission via viral RNA positive blood and/or the potential therapeutic value of convalescent plasma. (2) Methods: A total of 300 blood samples were sequentially collected from unvaccinated donors who donated blood to the blood bank of Prince Mutaib Bin Abdulaziz Hospital in Sakaka, Al-Jouf, Saudi Arabia. Specific ELISA was used to detect anti-SARS-CoV-2 IgG and IgM antibodies. SARS-CoV-2 was detected using specific real-time reverse-transcription PCR (rRT-PCR). (3) Results: The prevalence of anti-SARS-CoV-2 IgG was low (9%), whereas the prevalence of anti-SARS-CoV-2 IgM was high (65%). Relevant demographics, anthropometrics, and lifestyle factors revealed significant associations (*p* < 0.05) between IgM-positivity only vs. age (age group 21–30 years), postgraduate education, no history of international travel, IgG-negativity, and absence of experience with COVID-19-like symptoms. Furthermore, there are significant associations (*p* < 0.05) between IgG-positivity only vs. age (age group 21–30 years), postgraduate education, and being a non-healthcare worker. All donors in the anti-SARS-CoV-2 IgG-positive group (*n* = 27) had previously experienced symptoms similar to COVID-19 (*p* < 0.001) and most of them (*n* = 24) showed anti-SARS-CoV-2 IgM-positive test (*p* = 0.006). However, all the samples tested negative for SARS-CoV-2 RNA using rRT-PCR. (4) Conclusion: Our findings add to the growing body of evidence that donated blood is safe, with the added benefit of convalescent plasma rich in potentially neutralizing IgG and IgM against SARS-CoV-2.

## 1. Introduction

On 27 December 2019, the coronavirus disease-19 (COVID-19), caused by the severe acute respiratory syndrome-coronavirus-2 (SARS-CoV-2) in Wuhan, China, was discovered and spread to the rest of the world [1]. Clinical manifestations of COVID-19 range from severe pneumonia with respiratory distress (mostly in elderly people with underlying comorbidities) to mild clinical manifestations or asymptomatic carriage (mostly in children and young adults) [2].

Asymptomatic cases pose an infection control challenge and may aid in the spread of SARS-CoV-2. COVID-19 infection was reported to have been transmitted from an asymptomatic case in Germany [3]. In Wuhan, an asymptomatic 10-year-old boy who tested positive for SARS-CoV-2 via real-time reverse-transcription PCR (rRT-PCR) and CT-scan was suspected of being the source of infection for all of his family members who tested positive [4].

Although SARS-CoV-2 is primarily a respiratory virus, the possibility of transmission via other body fluids such as semen [5], urine [6], Saliva [7], tears and conjunctival secretions [8] are being investigated. Coronavirus RNA can be detected in blood lymphocytes and plasma, raising the possibility of virus transmission via blood transfusion. Many blood banks in China have a checklist for the following points before blood donation during the current pandemic: (1) taking the donor’s body temperature, (2) determining whether the donor or relatives have respiratory symptoms, have recently traveled to high-risk areas within the last 28 days, or are at high risk, (3) asking all blood donors about their physical condition after donation, and (4) removing un-transfused blood products from asymptomatic infected donors [9].

During the SARS-CoV-2 pandemic, the entire world faced several unknowns, and stricter measures, such as testing donated blood for SARS-CoV-2 RNA and antiviral antibodies, or the use of pathogen reduction/inactivation technologies that aim to eradicate or reduce the potential risk of coronavirus transmission through blood or blood products, were implemented when necessary. Wuhan blood banks have been conducting SARS-CoV-2 RNA blood testing since 10 February 2020 [10]. Blood transfusion biosafety in laboratories and blood banks should be improved during pandemics. Because 40–45% of COVID-19 cases are asymptomatic, blood donation safety should be taken into account [11,12,13]. Furthermore, there is a risk of SARS-CoV-2 reactivation [14,15]. Asymptomatic and reactivated COVID-19 cases may be eligible for blood donation.

Previous outbreaks of many emerging viruses in China hurt blood donation and transfusion [16]. The viral RNA was detected by PCR in the serum or plasma of SARS-CoV [17,18,19,20], MERS-CoV [21], or SARS-CoV-2 patients [22] within varying time frames after the symptoms appeared. Although the WHO reported in 2003 that no SARS-CoV cases had been detected through blood transfusion, there is still a theoretical risk of SARS-CoV transfusion transmission [23].

There is currently insufficient data on the potential role of donated blood in SARS-CoV-2 transmission. To ensure public safety, a better understanding of the prevalence of SARS-CoV-2 transmission via blood and blood products is required. This is the first study in Saudi Arabia to assess the pre-vaccination era donated blood content of specific anti-SARS-CoV-2 antibodies as evidence for convalescent plasma therapeutic use, as well as blood-borne transmission safety as confirmed by rRT-PCR viral RNA testing, and its relationship to relevant demographics, anthropometrics, and lifestyle.

## 2. Materials and Methods

### 2.1. Study Design, Data, and Samples Collection

The Local Committee for Ethics of Scientific Research, Jouf University, Sakaka, Saudi Arabia, granted bioethical approval (#26-06/42). After securing the bioethical approval, 300 blood samples were collected during the following three-month period in a cross-sectional study. The recruited donors were sequentially included in the random selection of samples from the donated blood consecutively received in the blood bank of Prince Mutaib Bin Abdulaziz Hospital, Sakaka. Each sample (5 mL) was collected in a sterile EDTA-containing tube and transported in an icebox to the Microbiology and Immunology Laboratory at Jouf University’s College of Medicine for further processing.

The data of the participating blood donors were reviewed for previously experienced symptoms similar to COVID-19, having a previous SARS-CoV-2 RT-PCR-positive or negative diagnosis, coming into contact with a person who was SARS-CoV-2 RT-PCR-positive, coming into contact with a person who was suffering from symptoms similar to COVID-19 but did not have SARS-CoV-2 RT-PCR or had negative-SARS-CoV-2 RT-PCR. The COVID-19 infection liability relevant demographic, anthropometric, and lifestyle characteristics of blood donors were recorded, including age, gender, education level, occupation (medical vs. non-medical), physical activity (daily activity sedentary style vs. regular physical exercising), body mass index (BMI, kg/m^2^), smoking, traveling abroad, vitamin supplement (no vs. on regular supplementation), and diet/beverage (healthy balanced diet/regular herbal beverages vs. unhealthy dieting/no herbal beverages).

### 2.2. Immunological Detection of Antibodies

To avoid contamination, the samples were processed aseptically upon arrival at the Microbiology and Immunology Laboratory. After 10–20 min at room temperature, blood samples were centrifuged for 10 min at 3,000 rpm. The plasma supernatant was aliquotted and kept at –80 °C [24]. Anti-SARS-CoV-2 antibodies (IgM and IgG) were detected immunologically using sensitive (>95%) and specific (>95%) ELISA kits (SunLong Biotech Co., LTD., Hangzhou Zhejiang, China).

### 2.3. SARS-CoV-2 RNA Detection

Because the nucleic acids of RNA viruses are unstable and may lose their stability when subjected to repeated freezing and thawing, a fresh plasma aliquot was used. RT-PCR is the most widely used and effective method for detecting pathogenic viruses in blood. RNA was extracted from plasma samples using commercially available kits (QIAamp^®^ Viral RNA Mini Kit, QIAGEN^®^, Hilden, Germany) as instructed. TaqMan probe real-time fluorescent RT-PCR amplification of SARS-CoV-2 *envelope protein* (*E*) and *RNA-dependent RNA polymerase* (*RdRp*) gene fragments using specific kits (The 1copyTM COVID-19 qPCR Multi-Kit, 1 Drop Inc., Gyeonggi-do, Republic of Korea) was used to test the presence of SARS-CoV-2 RNA in the samples. The SARS-CoV-2 primer and probe sets were created following the “World Health Organization interim guidance for laboratory testing for 2019 novel coronavirus (2019-nCoV) in humans”. Following extraction, the purified RNA was reverse-transcribed into cDNA using reverse transcriptase and then amplified using Taq DNA polymerase in the RT-PCR instrument (CFX96TM Real-Time PCR Detection System, Bio-Rad, Inc., California, USA). Control 1 (*E* gene plasmid) and control 2 (*RdRp* gene plasmid) were used as positive controls. Texas Red channel detection of internal positive control (human *GAPDH* gene) was used. Negative control was included in the reaction (DW NTC; no template control). The amplification conditions were as follows: 10 min at 55 °C for reverse transcription, 3 min at 95 °C, then 45 cycles of 15 s at 95 °C and 30 s at 58 °C, as previously mentioned [25]. A cycle threshold value (Ct-value) of ≤40 was considered a valid (+) test, and a Ct-value of >40 was considered an invalid (−) test.

### 2.4. Data Analysis

Data were fed to the computer and analyzed using IBM SPSS software package version 20.0. (Armonk, NY, USA: IBM Corp). Categorical data were represented as numbers and percentages. The Chi-square test was applied to investigate the association between the categorical variables. Alternatively, Fisher’s Exact or Monte Carlo correction test was applied when more than 20% of the cells have an expected count of less than 5. For continuous data, they were tested for normality by the Kolmogorov- Smirnov and Shapiro-Wilk test. Quantitative data were expressed as a range (minimum and maximum), mean, standard deviation, and median. A student *t*-test was used to compare two groups for normally distributed quantitative variables. The significance of the obtained results was determined by using a *p*-value of ≤0.05.

## 3. Results

The results of the study including the demographic, anthropometric, and lifestyle characteristics of blood donors in relation to anti-SARS-CoV-2 IgM and IgG detected are summarized in Table 1. The data revealed that 19% (*n* = 57/300) of the participants had previously experienced symptoms similar to COVID-19, but they had no previous SARS-CoV-2 RT-PCR positive or negative diagnosis or had come into contact with someone who was SARS-CoV-2 RT-PCR-positive. All of the blood donors were concerned about coming into contact with someone who had symptoms similar to COVID-19 with/without SARS-CoV-2 RT-PCR testing. It is worth noting that the COVID-19 vaccines had not yet been instituted in Saudi Arabia at the time of the study, so all blood donors received no dose of the COVID-19 vaccines.

In the tested donated blood samples, there was a low prevalence (9%) (*n* = 27/300) of anti-SARS-CoV-2 IgG and a high prevalence (65%) (*n* = 195/300) of anti-SARS-CoV-2 IgM.

There are statistically significant (*p* < 0.05) associations between anti-SARS-CoV-2 IgM-positivity and age (where anti-SARS-CoV-2 IgM-positivity was more prevalent in the age group 21–30 years), education (where anti-SARS-CoV-2 IgM-positivity was more prevalent in people with postgraduate studies), travel history (where anti-SARS-CoV-2 IgM-positivity was more prevalent in people with no travel history), and the previous experience of symptoms similar to those of COVID-19 (where anti-SARS-CoV-2 IgM-positivity was more prevalent in participants who previously did not experience symptoms similar to those of COVID-19).

There are statistically significant (*p* < 0.05) associations between anti-SARS-CoV-2 IgG-positivity and age (where anti-SARS-CoV-2 IgG-positivity was more prevalent in the age group 21–30 years), education (where anti-SARS-CoV-2 IgG-positivity was more prevalent in people with postgraduate studies) and occupation (where anti-SARS-CoV-2 IgG-positivity was more prevalent in non-healthcare workers). All donors in the anti-SARS-CoV-2 IgG-positive group (*n* = 27) had previously experienced symptoms similar to COVID-19 (*p* < 0.001). Furthermore, among the anti-SARS-CoV-2 IgG-positive group, 24 (88.9%) were positive for anti-SARS-CoV-2 IgM, while 3 (11.1%) were negative (statistically significant association, *p* = 0.006).

The rRT-PCR results showed that all of the donated blood samples tested negative for SARS-CoV-2 RNA.

## 4. Discussion

Worldwide, as of 27 May 2022, there have been 525,467,084 COVID-19 confirmed cases, including 6,285,171 deaths, reported to the WHO [26]. Although coronaviruses typically infect the respiratory tract, viral shedding in serum or plasma is to be expected. This raises concerns about the safety of donated blood products, because it is primarily dependent on voluntary blood donors [27,28]. Given the increasing number of asymptomatic [11,12,13] and reactivated SARS-CoV-2 cases [14,15], blood safety and coronaviruses should be prioritized, particularly in high-risk areas [10]. Although blood transfusion is an important part of the health care system, the adequate and safe availability of blood products to meet patients’ needs became a major concern during the COVID-19 pandemic [29]. Generally, it is believed that it is a key time to focus on asymptomatic (with no symptoms) and oligosymptomatic (with so mild symptoms that remain unrecognized) COVID-19 patients as they can be silent reservoirs to circulate SARS-CoV-2 infections [30].

Data on the potential role of donated blood in SARS-CoV-2 transmission are limited, and this is the first study in Saudi Arabia to evaluate donated blood as a potential source of SARS-CoV-2 transmission. We used sensitive and specific ELISA and rRT-PCR assays to test 300 blood samples collected from COVID-19-unvaccinated healthy blood donors in Sakaka, Al-Jouf, Saudi Arabia for anti-SARS-CoV-2 immunoglobulins and SARS-CoV-2 RNA. Despite the fact that all of the blood donors did not receive COVID-19 vaccinations, as the time was pre-vaccination, the tested blood samples were positive for anti-SARS-CoV-2 IgM and anti-SARS-CoV-2 IgG at rates of 65% and 9%, respectively. Furthermore, 8% (*n* = 24/300) of the blood samples tested positive for both anti-SARS-CoV-2 IgM and anti-SARS-CoV-2 IgG. The rRT-PCR is the gold standard for the diagnosis of COVID-19, but it is relatively expensive and requires standardized laboratories and trained operators. During a pandemic, many cases may be asymptomatic or afford their prolonged and/or progressive symptoms before seeking medical care. Thus, serologic testing, being cheaper and easier than the rRT-PCR, can help in picking up many of these patients instead of the standard molecular technique [31]. The current study’s findings confirm the notion that the true extent of the COVID-19 burden may be underestimated, and improved serological detection of specific SARS-CoV-2 immunoglobulins may aid in estimating the true rates of SARS-CoV-2 infections, especially where rRT-PCR is not available, a key point for effective planning and implementation of efficient infection prevention and control strategies.

Furthermore, the predominance of anti-SARS-CoV-2 IgM in the tested samples suggests that the majority of the participants were recently infected with SARS-CoV-2, despite the strict infection prevention and control measures in place at the time. This adds to the evidence that widespread COVID-19 vaccination is necessary to reduce the number of infected people who appear healthy and can donate blood.

Despite being positive for anti-SARS-CoV-2 immunoglobulins, 81% (*n* = 243/300) of the participants in the study did not previously experience symptoms similar to those of COVID-19. Many studies have reported asymptomatic SARS-CoV-2 exposure with the generation of anti-SARS-CoV-2 immunoglobulins. For example, it was reported that the overall seroprevalence of anti-SARS-CoV-2 immunoglobulins was 10.6% [32] and 24.4% [33] among asymptomatic participants. Long and his colleagues discovered that asymptomatic SARS-CoV-2 infected people have anti-SARS-CoV-2 IgM and IgG antibodies. However, IgG levels in the asymptomatic group were significantly lower than in the symptomatic group. Furthermore, the researchers reported that a significant proportion of the IgG-positive group became seronegative with time. Likewise, symptomatic patients had a decrease in IgG levels two to three months after infection. The weaker immune response in asymptomatic individuals raises concerns about the acquired immunity and serological surveys [34]. This contradicted previous research on SARS-CoV or MERS-CoV, which found that IgG levels remained elevated for at least a year after infection [35,36].

Thirty people (52.6%) were anti-SARS-CoV-2 IgG-negative among the participating blood donors who had previously experienced symptoms similar to those of COVID-19 (*n* = 57). This can be explained by either the fading of anti-SARS-CoV-2 IgG after 2-3 months of exposure [34], or the misinterpretation of symptoms as being related to COVID-19, especially unconfirmed with a previous SARS-CoV-2-RT-PCR-positive diagnosis, or come into contact with someone who was SARS-CoV-2 RT-PCR-positive. Some researchers studied the dynamics of neutralizing antibody titers in COVID-19 convalescent patients and discovered a drop in immunoglobulin levels 6–7 weeks after infection [37]. Mathematical modeling, on the other hand, inferred a short duration of immunity after SARS-CoV-2 infection [38]. The decrease in IgG levels sheds light on the inability to use COVID-19 ‘immunity passports’ and suggests that public health interventions such as social distancing, isolation of infected persons and their contacts, and widespread use of COVID-19 vaccine booster doses be extended.

Fortunately, the current study’s findings show that donated blood is safe and contains anti-COVID-19 immunoglobulins, which support the use of convalescent plasma as adjuvant therapy during the treatment of severely ill patients. This should be viewed cautiously in light of the virus’s many emerging variants and for our antibodies not being tested for their neutralizing ability.

The rRT-PCR analysis revealed that all of the tested donated blood samples were negative for SARS-CoV-2 RNA in the current study. This is consistent with two recent studies [27,28]. Owusu and his colleagues tested plasma from SARS-CoV-2 infected individuals in Ghana, but they found SARS-CoV-2 viral RNA in only 1.03% of the tested plasma samples, concluding that blood transfusion is negligible risk factor for SARS-CoV-2 transmission [27]. A systematic review and meta-analysis also revealed that SARS-CoV-2 poses no direct threat to blood safety [28]. On the other hand, SARS-CoV-2 RNA has been found in the blood at varying levels in some published studies [39,40]. Wang and his colleagues discovered SARS-CoV-2 RNA in three of 307 blood samples (1%) [39]. Wei and his colleagues investigated 15 patients (three of whom had severe COVID-19 presentation) and found SARS-CoV-2 RNA in the blood of six patients (40%), two of whom had severe COVID-19 presentation. The RNA was detected in both cases in whole blood and serum [40]. Furthermore, Chang and co-investigators suspected that the risk of SARS-CoV-2 transmission through blood transfusion is higher than that of other coronaviruses, necessitating careful evaluation of multiple measures and procedures such as postponement of donation, screening for virus-related antibodies, testing for SARS-CoV-2 RNA, or use of pathogen inactivation/reduction technologies [10].

The American Association of Blood Banks (AABB) [41] and the European Center for Disease Prevention and Control (ECDC) [42] published guidelines for rapid risk assessments of blood safety during the SARS-CoV-2 outbreak in January 2020. The AABB’s Transfusion Transmitted Diseases (TTD) Committee expressed support for a variety of approaches, including but not limited to voluntary implementation of travel deferrals (defer prospective donors for 28 days after their exit from China or other severely affected countries, the 28-day period covers twice the maximum incubation period of SARS-CoV-2), as well as a combination of deferrals related to illness and contact, as well as enhanced education (a more aggressive set of interventions regarding blood collection in severely affected areas, similar to those implemented during the SARS outbreak, including a combination of travel deferrals, deferrals for contact with SARS, and deferrals for a SARS diagnosis, with enhanced donor education) [41]. Furthermore, the ECDC advised any person returning from China, or any confirmed case to postpone blood and cell donation for 21 days after probable exposure, and deferral of recovering confirmed COVID-19 cases for at least 28 days after therapy completion and symptom resolution [42].

Finally, it is important to note that the presence of SARS-CoV-2 RNA in the blood does not imply infectiousness. According to our findings, the safety of blood transfusion and the critical role of immunoglobulins in convalescent plasma outweigh the potential risks. Although our RT-PCR targeted genes that are rarely affected by mutations, our study’s limitations include a large number of emergent virus variants, and not testing the neutralizing potential of the detected antibodies.

## 5. Conclusions and Recommendations

For the first time in Saudi Arabia, we assessed the safety of donated blood as a possible source of SARS-CoV-2 transmission, a source of anti-virus antibodies, and infection-relevant demographic, anthropometric, and lifestyle risk factors. Our pre-vaccination blood samples revealed a high prevalence of anti-SARS-CoV-2 IgM, which supports the therapeutic benefit of convalescent plasma, though only after testing its neutralizing ability. By the rRT-PCR, all samples were found to be negative for SARS-CoV-2 RNA. A limitation of our findings is the large number of emerging variants that could have escaped detection. These findings back up previous reports about the safety of blood transfusions during the current COVID-19 pandemic. Larger studies with vaccinated blood donors are needed to assess the durability of the anti-SARS-CoV-2 immunoglobulins.

## Figures and Tables

**Table 1:** Comparison of the demographics, lifestyle, and anthropometrics of blood donors stratified for the extent of anti-SARS-CoV-2 IgG or anti-SARS-CoV-2 IgM in their plasma (Total *n* = 300). Data shown are frequencies; *n* (%), and mean ± SD (median; range).

Characteristic		Distribution (*n* = 300)	Anti-SARS-CoV-2 IgG		Test of Significance	*p*	Anti-SARS-CoV-2 IgM		Test of Significance	*p*
			−ve (*n* = 273)	+ve (*n* = 27)			−ve (*n* = 105)	−ve (*n* = 195)		
Age; Years	≤20	12 (4.0%)	12 (4.4%)	0(0.0%)	χ^2^ = 8.273 *	^MC^*p* = 0.034 *	6 (5.7%)	6(3.1%)	χ^2^ = 9.275^*^	0.026 *
	21–30	150 (50.0%)	132 (48.4%)	18(66.7%)			63 (60.0%)	87(44.6%)		
	31–40	90 (30.0%)	81 (29.7%)	9(33.3%)			24 (22.9%)	66(33.8%)		
	41–45	48 (16.0%)	48 (17.6%)	0(0.0%)			12 (11.4%)	36(18.5%)		
Gender	Male	255 (85.0%)	231 (84.6%)	24 (88.9%)	χ^2^ = 0.352	^FE^*p* = 0.778	87 (82.9%)	168(86.2%)	χ^2^ = 0.582	0.446
	Female	45 (15.0%)	42 (15.4%)	3 (11.1%)			18 (17.1%)	27(13.8%)		
Education	Pre-university degree	36 (12.0%)	36 (13.2%)	0 (0.0%)	χ^2^ = 10.161 *	^MC^*p* = 0.013 *	18 (17.1%)	18(9.2%)	χ^2^ = 22.461 *	<0.001 *
	University student	93 (31.0%)	84 (30.8%)	9 (33.3%)			45 (42.9%)	48(24.6%)		
	Bachelor	45 (15.0%)	36 (13.2%)	9 (33.3%)			6 (5.7%)	39(20.0%)		
	Postgraduates	126 (42.0%)	117 (42.9%)	9 (33.3%)			36 (34.3%)	90(46.2%)		
Occupation	M/AHS	15 (5.0%)	12 (4.4%)	3 (11.1%)	χ^2^ = 6.482 *	^MC^*p* = 0.023 *	9 (8.6%)	6(3.1%)	χ^2^ = 5.492	0.064
	HCW	36 (12.0%)	36 (13.2%)	0 (0.0%)			15 (14.3%)	21(10.8%)		
	NHCW	249 (83.0%)	225 (82.4%)	24 (88.9%)			81 (77.1%)	168(86.2%)		
BMI (kg/m^2^)	Mean ± SD	25.33 ± 2.82	25.35 ± 2.85	25.11 ± 2.47	t = 0.422	0.673	25.11 ± 2.42	25.45 ± 3.01	t = 0.972	0.332
	Median (Min.–Max.)	26 (19–41)	26 (19–41)	26 (20–28)			25 (20–31)	26 (19–41)		
Travel history	No	264 (88.0%)	237 (86.8%)	27 (100.0%)	χ^2^ = 4.046	^FE^*p* = 0.056	78 (74.3%)	186 (95.4%)	χ^2^ = 28.771 *	<0.001 *
	Yes	36 (12.0%)	36 (13.2%)	0 (0.0%)			27 (25.7%)	9 (4.6%)		
IgM positivity	No	105 (35.0%)	102 (37.4%)	3 (11.1%)	χ2 = 7.443 *	0.006 *				
	Yes	195 (65.0%)	171 (62.6%)	24 (88.9%)						
IgG positivity	No	273 (91.0%)					102 (97.1%)	171 (87.7%)	χ^2^ = 7.443 *	0.006 *
	Yes	27 (9.0%)					3 (2.9%)	24 (12.3%)		
Previous experience of COVID-19-like symptoms	No	243 (81.0%)	273 (100.0%)	0 (0.0%)	–	–	77 (73.3 %)	166 (85.1%)	χ^2^ = 5.42 *	0.021 *
	Yes	57 (19.0%)	0 (0.0%)	27 (100.0%)			28 (26.7%)	29 (14.9%)		
Smoking	No	261 (87.0%)	237 (86.8%)	24 (88.9%)	χ^2^ = 0.094	^FE^*p* = 1.000	90 (85.7%)	171 (87.7%)	χ^2^ = 0.236	0.627
	Yes	39 (13.0%)	36 (13.2%)	3 (11.1%)			15 (14.3%)	24 (12.3%)		
Physical activity	No	267 (89.0%)	243 (89.0%)	24 (88.9%)	χ^2^ = 0.000	^FE^*p* = 1.000	90 (85.7%)	177 (90.8%)	χ^2^ = 1.781	0.182
	Yes	33 (11.0%)	30 (11.0%)	3 (11.1%)			15 (14.3%)	18 (9.2%)		
Diet/beverages	No	261 (87.0%)	237 (86.8%)	24 (88.9%)	χ^2^ = 0.094	^FE^*p* = 1.000	90 (85.7)	171 (87.7)	χ^2^ = 0.236	0.627
	Yes	39 (13.0%)	36 (13.2%)	3 (11.1%)			15(14.3%)	24 (12.3)		
Vitamin supplements	No	270 (90.0%)	246 (90.1%)	24 (88.9%)	χ^2^ = 0.041	^FE^*p* = 0.741	90 (85.7%)	180 (92.3%)	χ^2^ = 3.297	0.069
	Yes	30 (10.0%)	27 (9.9%)	3 (11.1%)			15 (14.3%)	15 (7.7%)		

−ve: Negative; +ve: Positive; BMI: Body mass index; COVID-19: Coronavirus disease-19; IgG: Immunoglobulin G; IgM: Immunoglobulin M; SARS-CoV-2: Severe acute respiratory syndrome-Coronavirus-2; M/AHS: Medical/Allied health student; HCW: Healthcare workers; NHCW: Non-Healthcare workers. χ^2^: Chi-square test; *t*: Student *t*-test; ^FE^: Fisher Exact; ^MC^: Monte Carlo. *p*: *p*-value for comparing between negative and positive IgG, or, between negative and positive IgM. *: Statistically significant at *p* ≤ 0.05; SD: Standard deviation from the mean.

## Data Availability

All data are available in the manuscript.
